# Internet Entrepreneurship Psychology for College Students and Internet Financial Crimes Prevention

**DOI:** 10.3389/fpsyg.2022.892061

**Published:** 2022-05-30

**Authors:** Ruihua Li

**Affiliations:** School of Law, Southeast University, Nanjing, China

**Keywords:** internet financing, student entrepreneurship, entrepreneurial psychology, internet financial crime, crime prevention

## Abstract

This work aims to study the entrepreneurial intention of college students’ Internet Entrepreneurship and the potential risk of Internet financial crime from a psychological perspective. Here, the relevant psychological theories are used to analyze the personal and social factors of College Students’ Internet Financing Entrepreneurship. Specifically, College Students’ Internet Financial Entrepreneurship factors are examined alongside the excellence and feasibility of Internet Entrepreneurship. Secondly, it introduces the main types of Internet financial crimes and analyzes the psychological traits of college students’ Internet financial crimes. Further, the research reveals the possibility of college students committing Internet financial crimes by investigating the current situation of College Students’ Internet Entrepreneurship through a questionnaire survey. The results show that 78% of the respondents have studied college Innovation and Entrepreneurship Education (IEE). More than 90% are interested in entrepreneurship, but most have not undertaken entrepreneurship. Therefore, the current college students’ enthusiasm for Internet Financial Entrepreneurship is affected by many factors and is not high. Finally, given the existing problems, hierarchical nesting prevention measures are proposed to prevent college students’ Internet financial crimes during entrepreneurship. This work provides a reference for analyzing the psychological factors of College Students’ Internet Financial Entrepreneurship and preventing potential Internet financial crimes.

## Introduction

The dynamic global economic environment has adjusted the global industrial structure, thus intensifying competition among industries. Under such a backdrop, college students feel pressure from employment difficulties (Ma et al., 2020). In particular, in the knowledge economy era and industrial transformation, entrepreneurship can create more employment opportunities and promote socio-economic development. Meanwhile, with advancing Internet technology, Internet entrepreneurship has spread quickly with characteristics like low cost, low risk, and low threshold (Wu and Wu, 2019). College Students are well aware that entrepreneurial activities are an important means of relieving employment pressure. However, without a comprehensive understanding of the relevant laws of Internet financing, college graduates have become the main driving force behind online misconducts, such as Internet fraud or privacy disclosure. Meanwhile, the current law system has no intention to claim college entrepreneurs as conducting an occupational crime but only blame their behaviors due to a specific Criminal Trait using the Internet. Therefore, it is necessary to analyze the College Students’ Internet Entrepreneurial Psychology, determine the risk of Internet financial crimes, and carry out effective prevention and control. Doing so can stabilize the form of College Students’ Internet entrepreneurship and the development of the Internet social economy. In response to the above problems, this work will explore the main factors affecting Internet Financing entrepreneurship and crime. College Students’ entrepreneurship will be mined in-depth from Trait Theory to practical cognition for the potential crime risk.

At present, scholars have conducted official research on Internet Financing entrepreneurship and the problem of Internet financial crime based on psychology. Barba-Sánchez et al. (2021) examined the impact of Smart City Construction (SCC) on the demand for high-skilled workers and the influence of younger generations on the Smart City job market. They explained the relationship between educational Decision-Making and Smart City entrepreneurial activity through a digital model. Barba-Sánchez et al. (2022) studied the College Students’ entrepreneurial intention factors. They confirmed that College Students’ high environmental protection awareness impacted their attitudes toward entrepreneurial behavior. Hua and Liu (2021) studied the intervention of psychology courses in Colleges and Universities on students’ psychological problems. They found that traditional psychology courses only focused on preventing and correcting students’ psychological problems, ignoring the care of students’ emotions. Obviously, the traditional manner was not conducive to cultivating students’ health psychological potential. They proposed an Internet education-oriented teaching model by combining the mixed teaching theory of psychology and entrepreneurship education. The Graph Convolutional Neural Network (GCNN) was used to predict students’ performance and learning status. Then, corresponding countermeasures were formulated to enhance students’ learning enthusiasm. Zhao et al. (2020) studied the influence of entrepreneurial Psychological Capital (PsyCap) on students’ entrepreneurial intentions. The research discovered that traditional capital was the direct factor driving entrepreneurial behavior. In comparison, PsyCap did not directly affect the entrepreneurial intention but through traditional capital. The Structural Equation Model (SEM) analysis showed that only financial, human capital, social capital, and PsyCap impacted students’ entrepreneurial intentions, and traditional entrepreneurial capital played an intermediary role. Brands and van Wilsem (2021) surveyed the level of fear of cyber-crime in the general population and the correlation between cyber-fear and avoidance behavior. According to the survey results, the level of fear of cybercrime among the Dutch population was moderate. With online shopping and business popularity, the fear of online financial crime hindered people’s perception of online freedom, further affecting socio-economic development. Fu and Zhao (2020) investigated the impact of Internet development on College Students’ entrepreneurship. They shared the impact of online entrepreneurship on College Students’ entrepreneurship. Based on the current situation, the main problems faced by College Students’ Internet entrepreneurship were analyzed, and corresponding improvement strategies were put forward.

This work studies the psychological factors of College Students’ Internet Financing Entrepreneurship, understands College Students’ deviation behavior during entrepreneurship, and the root cause of Internet Financing risks. As such, it pinpoints the criminal risks during College Students’ Internet Financing entrepreneurship and formulates corresponding risk prevention strategies. Specifically, the psychological factors of College Students’ Internet Financing Entrepreneurship are firstly analyzed. Secondly, combined with the laws of Internet Financing, it analyzes the criminal risks involved in College Students’ Internet Financial Entrepreneurship. It discusses the psychological factors of College Students’ Internet financial crimes. Finally, the financial crime prevention mechanism of College Students’ Internet Financial Entrepreneurship is explored by utilizing hierarchical nesting prevention. The research innovation lies in analyzing the potential criminal risks in the Internet Financial Entrepreneurship of College Students. The proposal provides a reference for preventing College Students’ Internet financial crimes.

## Psychoanalysis of College Students’ Entrepreneurship and Prevention of Internet Financial Crimes

### Psychoanalysis of College Students’ Internet Entrepreneurship

Information and network technology have changed the production pattern worldwide. It brings new market and entrepreneurial opportunities to the public, especially for college students facing increasing employment pressure. As a convenient and mature business model, Internet entrepreneurship lowers the threshold for students to start a business, attracting many college students to invest in Internet entrepreneurial activities (Krone et al., 2020). As one of many social activities, College Student Internet Entrepreneurship can only be guaranteed by cultivating students’ entrepreneurial ability (Liu et al., 2019; Miao, 2020). As a new entrepreneurial model, Internet entrepreneurship has the following characteristics:

1. Openness and sharing. Openness and sharing of information are the keynotes of Internet development. Venture enterprises can strengthen competitiveness by learning from successful enterprise models and developing soundly (Wu and Song, 2019). College student entrepreneurs can adopt a unique business model through the integration of fruitful models of supreme enterprises to improve the success rate (Shihua, 2020).
2. The threshold of entrepreneurship is low. The Internet has a wide range of coverage, and consumers and producers are closely connected through the Internet, reducing intermediate consumption in the industrial chain, expanding consumer groups, and obtaining more profits. With advanced IT, business information can be integrated, information acquisition costs can be reduced, and the financial market can be well judged.
3. Internet entrepreneurship has wide marketing channels (Liu, 2020). People have been interconnected through the Internet. Through chat groups, media, official accounts, and live broadcasts, College Student entrepreneurs can promote products, understand consumers’ actual needs, tighten enterprise-consumer relations, maintain good customer satisfaction, and obtain more profits.
4. Internet entrepreneurship is very convenient. To promote the enthusiasm of College Students entrepreneurship, many encouraging policies and measures have been laid down (Sun et al., 2020). The mature business model of venture enterprises can provide a practical business model for college students. The college and government entrepreneurship platform can provide financial, spatial, and legal help for college students, encouraging College Students’ Internet Entrepreneurship.

Many college students choose Internet entrepreneurship as their first step toward career development. Inevitably, both opportunities and pressure can be spotted in the Internet Financing market, which imposes a strict test on their psychological quality. To help students better cope with the social test, college Innovation and Entrepreneurship Education (IEE) must pay more attention to relieving College Students’ entrepreneurial psychological pressure and mental pressure by providing professional psychological support and help. The factors affecting college students’ entrepreneurship are mainly divided into personal and social factors. Entrepreneurial psychoanalysis analyzes students’ self-efficacy and personality traits. Particularly family, school, and gender factors must also be considered. Meanwhile, it is necessary to understand the dynamics of the Internet entrepreneurship environment and related policy support. Entrepreneurial intention is the main driving force for college students in the early stage of entrepreneurship. Accordingly, this work studies and analyzes the influencing factors of College Students’ entrepreneurial intention (Chang et al., 2020).

### College Students’ Internet Financial Crime Risks

With the development of Internet technology, the Internet has become indispensable in people’s production and life. The rich Internet resources have brought people a more convenient life and expanded the communication scope of financial activities. This, however, also has seeded many new crime types. Internet crime refers to the criminal behavior that endangers the system and content of an electronic information network or uses electronic information network technology to do social harm. It can be divided into electronic information Internet crime and information Internet-enabled crime (Hannah-Moffat, 2019). The increasing Internet crime cases have seriously harmed people’s life and property safety and disrupted social order. Among the initiators of Internet crime cases, College students, who master emerging network technologies merit in-depth research. Remarkably, College Students’ psychological problems must be expatiated to explore the main causes of Internet crimes. Based on this, corresponding crime prevention countermeasures can be formulated (Hecht et al., 2019).

Here are some of the typical Internet Financial crimes. (1) Raising funds through the Peer-To-Peer (P2P) lending network. The criminal subjects establish an investment and wealth management platform by purchasing packaged finished products. Then, they promise high returns to attract investors’ capital. Finally, when the time is right, they abscond with the collected money (Yang et al., 2019). (2) Advocating some frontier knowledge, such as Blockchain or digital currency. In this case, the subject introduces conceptual currencies without actual economic value to the public. It tricks them into investing through public opinion. (3) Confusing the concept of equity and stock. The subject makes the public believe that a company is about to go public. Then, they sell false funds and equity and other wealth management products as the so-called original shares by advocating excess returns. In this way, they accumulate a huge amount of wealth. (4) Using online shopping malls to encourage investors to recharge in the form of rebates and points. In this scenario, the subject sells huge numbers of unqualified products to fund illegally, bypassing the law-forbidden online pyramid selling (Erin et al., 2020). (5) Conducting futures trading by building an illegal platform. The subject will make profits by collecting margin, handling fees, or interest during the company’s operation.

Contact and violent crimes have gradually transformed into non-contact crimes in cyberspace. The Internet crime of College Students is mainly manifested as follows. (1) College Students use substantial network technology to invade personal online space and steal their online property or other interests. A few are influenced by hacker culture to satisfy their curiosity (Wang et al., 2021). (2) College Student uses online platforms to gain economic benefits by opening casinos, selling online pornography, and telecommunication fraud. In this part, College Students master new network technologies and equipment. Therefore, the number of College Students involved in such crimes is large and worth extensive attention (Reyns, 2019). (3) The purposes of College Student Internet crimes are various. Due to the virtual nature of cyberspace, it is difficult to target criminal subjects, especially Internet crimes committed by College Students. They have mastered some advanced network technology. The result is damage to national security and public social order.

### College Students’ Internet Technology-Based Criminal Psychology

From the perspective of criminology, the essence of college students’ Internet crime is to commit crime through IT. Internet criminal behaviors are more harmful than traditional ones. In some cases, this harmful consequence might have come from the positive behavior of the criminals who have successfully escaped from the law as their psychological expectation. Therefore, it is of great significance to study the psychological mechanism of college students’ Internet crime, and the mechanism should be analyzed from the following aspects.

1. The enlargement of profit-seeking psychology in the network space (Paat and Markham, 2021). The importance of wealth has been realized by College Students as they grow. Influenced by greedy psychology, College Students easily get frustrated with their current lives through comparison with their contemporaries, and they want more than what they can afford. Gradually, this may lead to some psychological problems. The popularization of IT has made it possible for College Students to accumulate wealth rapidly. For example, the latest IT has cut down information acquisition costs dramatically, and to get enormous profits, some College Students choose to steal and trade valuable information or publish false information to conduct frauds or raise illegal funds.
2. The fluke psychology fostered by Internet technology (Fansher and Randa, 2019). Most criminals will be punished for their illegal acts. While College Students who have committed Internet crimes may get away with it because Internet crime is intangible and hard to be found, and laws on Internet are imperfect. This has become the psychological root of College Students’ Internet crime. Meanwhile, College Students are more likely to commit Internet crimes to seek profit. IT, being a well-disguised technology, has greatly reduced people’s sense of guilt in criminal actions. This is true in the case of College Students who obtain profits through the dissemination of pornographic videos.
  Moreover, criminal evidence can be easily destroyed in Internet crime, making it more difficult to investigate and contribute to the arrogance of criminal behavior. For example, phone numbers can be hidden and transfer records can be eliminated through IT in telecommunication fraud, so no effective criminal punishment mechanism can be formed. In IT criminal behaviors, the reward is much more than the effort, so college students with fluke psychology are often involved. Besides, the concealment of Internet crime gives a mental hint of fluke psychology to College Students who commit Internet crime through high-tech means.
3. Many anti-social personalities can be displayed in cyberspace. In China, most students have to endure years of painstaking and boring school works, and they are often strictly supervised by their parents in their high school years. Thus, college may mean heaven for freedom for those students, when they are not properly guided, they may feel much depressed and get lonely because of sudden isolation from home and familiar school environment. Meanwhile, some of them may get addicted to online games and will be absorbed by the virtual world, and these students can hardly get approval from both school and society, losing their self-esteem and developing some psychological problems. Scholars believe these can contribute to most College Students’ deviant behaviors and criminal acts. Consequently, some students resort to the virtual world to express their hearty feelings, and some may get to the wrong way by exerting their negative emotions relentlessly or vent their uncontentious without control. By and by some of them may commit Internet crimes through IT and their command of knowledge.
4. The alienation of the curiosity psychology brought by cyberspace. College Students are in an important stage of physical and mental development, so they have a strong curiosity for the world. As the central way to connect the real world and the virtual world, the Internet has become the best place for College Students to seek novelty. But the information on the Internet is miscellaneous, and immature college students are vulnerable to the influence of pornography and violence. The curiosity psychology of hunting new things gradually changes into beauty-seeking psychology and deteriorates into anti-social crimes. In many ways, College Students’ Internet crime is mainly caused by psychological alienation caused by the adverse influence of Internet information.

### Questionnaire Design for College Students’ Internet Financial Entrepreneurship

Summarizing domestic and foreign research suggests that in the macro aspect, students’ entrepreneurial intention is mainly reflected in personal circumstances, physical fitness, and the external environment. The main index can be subdivided into secondary indexes: gender, age, education, risk-taking ability, achievement needs, and local culture. Then, all factors comprehensively contribute to students’ Entrepreneurial Psychology (Howard et al., 2019; Ruankaew, 2019). In particular, this work considers individual, family, and social factors to explore the influence of different factors on College Students’ Entrepreneurial Psychology. In order to do so, a questionnaire survey is conducted on several colleges and universities in Xi’an. Specifically, the “Questionnaire Star” is used to design and distribute the questionnaires, and 451 valid questionnaires are collected. The distribution and recovery of questionnaires meet scientific research needs (Musyoka et al., 2021). The survey focuses on in-campus College Students to better reflect the situation of College Students’ Internet entrepreneurship. The questionnaire is divided into two levels of evaluation indexes. The indexes selected here are the most important ones evaluated by ten experts. The questionnaire form is listed in Table 1.

**Table 1:** Questionnaire form for College Students’ Internet entrepreneurial intention.

Target	Primary index	Secondary index
QS of College Students’ Internet entrepreneurial intention	Personal status	Gender
		Age
		Major
		Grade
		Training experience in entrepreneurship education
		Internet entrepreneurship experience
	Social environment	Family condition
		School entrepreneurial atmosphere
		Regional policy support
		Financing channels
		Internet usage
		Social practices
		Understanding of the Internet and financial market development
	Personality	Challenge
		Cooperation
		Initiative
		Innovation

## Results Analysis

### Questionnaire Reliability and Validity Test

Statistical Packages for Social Sciences (SPSS) 22.0 was used to statistically analyze the collected questionnaire data. Confirmatory Factor Analysis (CFA) was used to test the construct validity. Meanwhile, the Average Variance Extracted (AVE) method was used to test the discriminant validity, and Cronbach’s α coefficient was used to test the reliability of the scales. As a result, the Kaiser–Meyer–Olkin (KMO) value was 0.967, and the KMO values of the remaining secondary indexes were all above 0.7. Thus, the designed scale has a good test effect and is suitable for factor analysis. The AVE of each dimension is greater than the correlation coefficient between each dimension, namely, the square of the standardized correlation.

Therefore, the designed scales have discriminant validity among each dimension. The calculated Cronbach’s *α* coefficient of all dimensions is greater than the judgment standard of 0.7. This proves that the overall internal consistency of the scale data is high, the scale is stable, and the overall reliability is good, which can reach the scientific research need.

### Analysis of Survey Results

**Figure 1:**
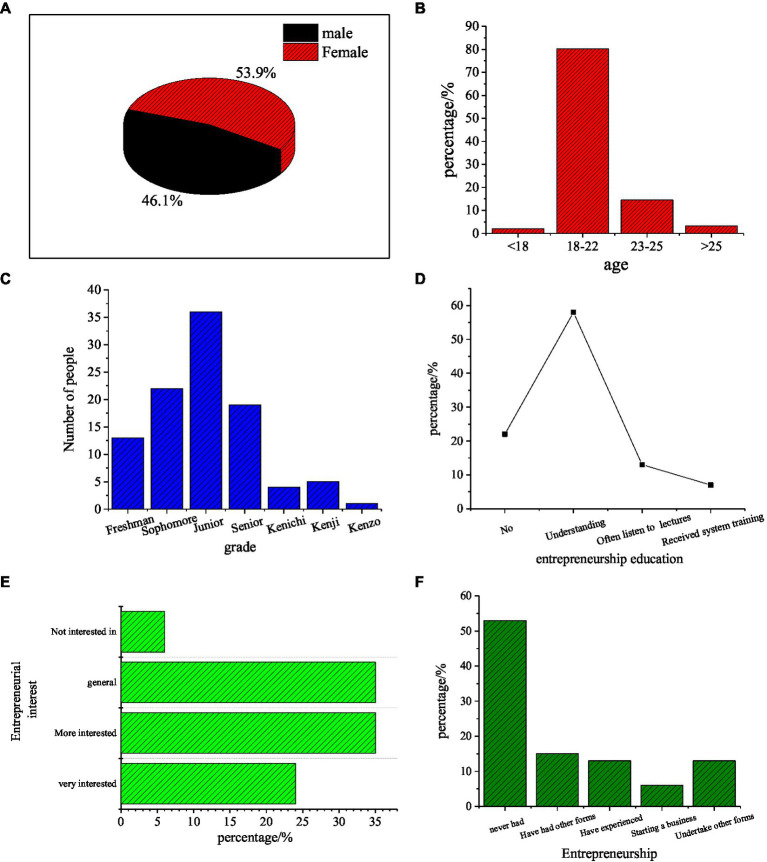
QS statistics. **(A)** Gender, **(B)** Age, **(C)** Grade, **(D)** IEE education, **(E)** Entrepreneurial intention, and **(F)** Entrepreneurship.

In order to study the entrepreneurial intention of college students, the statistical results of the survey respondents are shown in Figure 1.

As in Figure 1, the proportion of female students in the survey is slightly more than that of male students. The respondents are mostly 19- and 22-year-old undergraduates. First-year students account for 22%, and graduates account for no more than 10%. At the same time, 78% of students have studied college IEE, and more than 90% of students are interested in entrepreneurship. According to the analysis in Figure 1, students are full of interest in Internet Financial Entrepreneurship to improve their economic situations. However, 53% of the students have never engaged in actual entrepreneurial activities. The reasons are diverse, involving both personal and social factors. Most students never attempted to start an Internet business because of fear of being deceived.

### College Students’ Personal Traits Research

**Figure 2:**
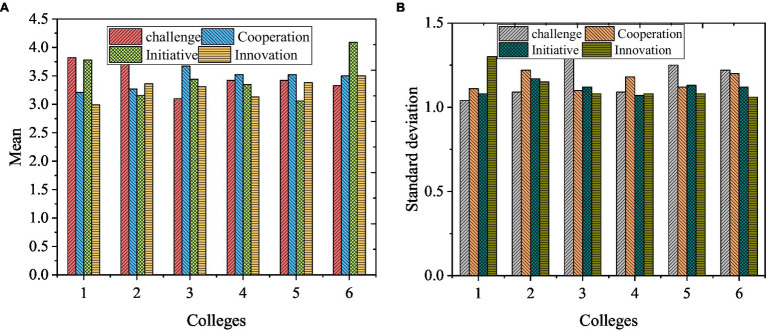
Description of personality. **(A)** Mean, and **(B)** Standard deviation.

The survey on college students’ personal traits considers challenge, cooperation, initiative, and innovation ability, evaluated by the 5-point scoring method. The results of the survey on six colleges are compared in Figure 2.

From Figure 2, the scores of personal traits of different colleges are different, and the mean value is concentrated around 3.50 points. Thus, the overall traits of the respondents are challenging, cooperative, proactive, and innovative. Surely, college students will encounter various problems during entrepreneurship. Mainly, these problems are caused by college students’ inability to integrate Internet technology into practical entrepreneurial activities effectively. As a result, they fail to build effective entrepreneurial ideas and programs and limited understanding of the financial market.

## Discussion

Due to the virtual nature of the Internet and the particularity of the Internet Financing market, ever more Internet financial crimes are emerging. With a semi-mature physical and mental state, College Students easily fall into a bad psychological state of profit-seeking, opportunism, or uncontrolled curiousness and get lost. As a result, the virtual world of the Internet may amplify their anti-social traits (Piquero et al., 2021). College Student entrepreneurship is carried out under opportunism and profit. Thus, it is more likely to be driven by various psychological factors, sometimes resulting in deviant or criminal behavior. Therefore, given the above College Students’ Internet crime behavior, it is necessary to establish hierarchically nested preventive measures to effectively guide and alleviate college students’ psychological problems and avoid Internet financial crimes (Gonçalves et al., 2020).

Therefore, it is necessary to comprehensively guide students’ entrepreneurial behavior, cultivate a healthy Internet entrepreneurial psychology, and control their behavior in entrepreneurial activities. Finally, the following suggestions are formulated for the prevention of College Students’ Internet crime:

1. It is necessary to cultivate the healthy psychology of College Students’ Internet entrepreneurship. Pay attention to the diversity of College Students’ Internet psychology, educate college students on the theory of self-control, and correct the psychological problems of Internet entrepreneurship. Only by doing so can a healthy Internet Financial Entrepreneurial Psychology be cultivated among college students (Baloran, 2020).
2. College IEE should strengthen guidance on College Students’ Internet Financing Entrepreneurship. It includes supporting guidelines, such as entrepreneurship guidance education, entrepreneurial psychological assessment, legal risk prediction, guidance on Internet development laws, and evolution of Internet Financing market laws (Cadenas et al., 2020; Wang and Chiou, 2020).
3. Government and relevant departments should strengthen the regulation of the Internet financial market and the guidance of College Students’ entrepreneurship. While standardizing the Internet Financing market, the government should reduce the social factors deviating College Students’ Internet Financial entrepreneurship. Meanwhile, schools should educate “social cognitive career theory” for College Students’ Internet Financial Entrepreneurship. As such, College Students’ entrepreneurial intentions will be fully explained to help them realize the uniqueness of Internet entrepreneurship as a career.

## Conclusion

In order to study the influencing factors of College Students’ Internet entrepreneurship, this work studies, based on the relevant psychological theories, the possible criminal behaviors during College Students’ entrepreneurship. Specifically, it analyzes the problems College Students may encounter during Internet Financial Entrepreneurship alongside the factors influencing Internet financial crimes. The proposed hypotheses are verified through statistics. The results show that personal and social factors affect College Students’ Internet Financial Entrepreneurial Psychology. Therefore, crime prevention measures must be carried out from College Students’ personal, college environment, and the Internet Financing market. Although some repeated claims in each countermeasure are repeated, they are essential for the field of College Students. Therefore, it puts forward the hierarchically nested prevention measure: “College Students-colleges—Internet Financing market.” The proposed measures can guide the psychology and entrepreneurial direction of College Students’ Internet Financial entrepreneurship and prevents Internet financial crimes. This concept can provide guidance for preventing College Students’ Internet financial crimes and help relevant colleges or departments better help College Students carry out entrepreneurial activities.

The research innovation is to analyze the Internet Financing Entrepreneurship and potential crime problems of College Students. However, there are still some deficiencies. The relevant evaluation indexes of the questionnaire are not comprehensive, and the research respondents are relatively concentrated. Therefore, the follow-up research will expand the research scope and strengthen the questionnaire form. The research fining provides a reference for preventing College Students’ Internet financial crimes.

## Data Availability Statement

The raw data supporting the conclusions of this article will be made available by the authors, without undue reservation.

## Ethics Statement

The studies involving human participants were reviewed and approved by Southeast University Ethics Committee. The patients/participants provided their written informed consent to participate in this study. Written informed consent was obtained from the individual(s) for the publication of any potentially identifiable images or data included in this article.

## Author Contributions

The author confirms being the sole contributor of this work and has approved it for publication.

## Conflict of Interest

The author declares that the research was conducted in the absence of any commercial or financial relationships that could be construed as a potential conflict of interest.

## Publisher’s Note

All claims expressed in this article are solely those of the authors and do not necessarily represent those of their affiliated organizations, or those of the publisher, the editors and the reviewers. Any product that may be evaluated in this article, or claim that may be made by its manufacturer, is not guaranteed or endorsed by the publisher.

## References

[ref1] Baloran E. T. (2020). Knowledge, attitudes, anxiety, and coping strategies of students during COVID-19 pandemic. J. Loss Trauma 25, 635–642. doi: 10.1080/15325024.2020.1769300

[ref2] Barba-Sánchez V. Mitre-Aranda M. del Brío-González J. (2022). The entrepreneurial intention of university students: an environmental perspective. Eur. Res. Manag. Bus. Econ. 28:100184. doi: 10.1016/j.iedeen.2021.100184

[ref3] Barba-Sánchez V. Orozco-Barbosa L. Arias-Antúnez E. (2021). On the impact of information technologies secondary-school capacity in business development: evidence From smart cities Around the world. Front. Psychol. 12:731443. doi: 10.3389/fpsyg.2021.731443, PMID: 34970182 PMC8712570

[ref4] Brands J. van Wilsem J. (2021). Connected and fearful? Exploring fear of online financial crime, internet behaviour and their relationship. Eur. J. Criminol. 18, 213–234. doi: 10.1177/1477370819839619

[ref5] Cadenas G. A. Cantú E. A. Lynn N. Spence T. Ruth A. (2020). A programmatic intervention to promote entrepreneurial self-efficacy, critical behavior, and technology readiness among underrepresented college students. J. Vocat. Behav. 116:103350. doi: 10.1016/j.jvb.2019.103350

[ref6] Chang S. H. Shu Y. Wang C. L. Chen M. Y. Ho W. S. (2020). Cyber-entrepreneurship as an innovative orientation: does positive thinking moderate the relationship between cyber-entrepreneurial self-efficacy and cyber-entrepreneurial intentions in non-IT students? Comput. Hum. Behav. 107:105975. doi: 10.1016/j.chb.2019.03.039

[ref7] Erin O. A. Kolawole A. D. Noah A. O. (2020). Risk governance and cybercrime: the hierarchical regression approach. Future Bus. J. 6, 1–15. doi: 10.1186/s43093-020-00020-1

[ref8] Fansher A. K. Randa R. (2019). Risky social media behaviors and the potential for victimization: a descriptive look at college students victimized by someone met online. Violence Gend. 6, 115–123. doi: 10.1089/vio.2017.0073

[ref9] Fu H. Zhao Y. (2020). Research on the current situation of college Students' online entrepreneurship. Int. Core J. Eng. 6, 275–280. doi: 10.6919/ICJE.202008_6(8).0037

[ref10] Gonçalves M. Monteiro I. Matos M. (2020). Trafficking in human beings: knowledge of Portuguese college students. J. Hum. Traffick. 6, 467–479. doi: 10.1080/23322705.2019.1631622

[ref11] Hannah-Moffat K. (2019). Algorithmic risk governance: big data analytics, race and information activism in criminal justice debates. Theor. Criminol. 23, 453–470. doi: 10.1177/1362480618763582

[ref12] Hecht B. Valaskova K. Kral P. Rowland Z. (2019). The digital governance of smart city networks: information technology-driven economy, citizen-centered big data, and sustainable urban development. Geopolit. Hist. Int. Relat. 11, 128–133. doi: 10.22381/GHIR111201910

[ref13] Howard R. M. Potter S. J. Guedj C. E. Moynihan M. M. (2019). Sexual violence victimization among community college students. J. Am. Coll. Heal. 67, 674–687. doi: 10.1080/07448481.2018.1500474 30257142

[ref14] Hua S. Liu F. (2021). A new hybrid teaching model for a psychology course. Int. J. Emerg. Technol. Learn. (iJET) 16, 206–219. doi: 10.3991/ijet.v16i03.20457

[ref15] Krone T. Spiranovic C. Prichard J. Watters P. Wortley R. Gelb K.. (2020). Child sexual abuse material in child-centred institutions: situational crime prevention approaches. J. Sex. Aggress. 26, 91–110. doi: 10.1080/13552600.2019.1705925

[ref16] Liu M. (2020). Relationship between psychological capital and employment area selection ability of college graduates: evidences from China. Rev. Argentina de Clín. Psicol. 29:468. doi: 10.24205/03276716.2020.63

[ref17] Liu X. Lin C. Zhao G. Zhao D. (2019). Research on the effects of entrepreneurial education and entrepreneurial self-efficacy on college students' entrepreneurial intention. Front. Psychol. 10:869. doi: 10.3389/fpsyg.2019.00869, PMID: 31068862 PMC6491517

[ref18] Ma L. Lan Z. Tan R. (2020). Influencing factors of innovation and entrepreneurship education based on the theory of planned behavior. Int. J. Emerg. Technol. Lear. (iJET) 15, 190–206. doi: 10.3991/ijet.v15i13.15345

[ref19] Miao Z. (2020). The influence factors of psychological understanding and behavior choice for legal industry entrepreneurs based on artificial intelligence technology. Front. Psychol. 11:1615. doi: 10.3389/fpsyg.2020.01615, PMID: 32793042 PMC7385309

[ref20] Musyoka C. M. Mbwayo A. Donovan D. M. Mathai M. (2021). mHealth-based peer mentoring for prevention of alcohol and substance abuse among first year university students: protocol for quasi-experimental intervention. J. Subst. Abus. 26, 53–59. doi: 10.1080/14659891.2020.1766131

[ref21] Paat Y. F. Markham C. (2021). Digital crime, trauma, and abuse: internet safety and cyber risks for adolescents and emerging adults in the 21st century. Soc. Work. Ment. Health 19, 18–40. doi: 10.1080/15332985.2020.1845281

[ref22] Piquero N. L. Piquero A. R. Gies S. Green B. Bobnis A. Velasquez E. (2021). Preventing identity theft: perspectives on technological solutions from industry insiders. Vict. Offenders 16, 444–463. doi: 10.1080/15564886.2020.1826023

[ref23] Reyns B. W. (2019). Online pursuit in the twilight zone: Cyberstalking perpetration by college students. Vict. Offenders 14, 183–198. doi: 10.1080/15564886.2018.1557092

[ref24] Ruankaew T. (2019). Employee theft among college students in the workforce. Int. Bus. Res. 12, 40–49. doi: 10.5539/ibr.v12n4p40

[ref25] Shihua L. (2020). Research on the psychological regulation mechanism of learning anxiety in English innovation and entrepreneurship in Minzu universities. Front. Educ. Res. 3:31315. doi: 10.25236/FER.2020.031315

[ref26] Sun D. Li S. Xu X. (2020). Analysis of reform and development strategies of China's internet innovation and entrepreneurship education. Entrep. Educ. 3, 77–93. doi: 10.1007/s41959-020-00024-6

[ref27] Wang Y. M. Chiou C. C. (2020). Factors influencing the willingness of Universities' business management departments to implement online entrepreneurship program and its effectiveness. Front. Psychol. 11:975. doi: 10.3389/fpsyg.2020.00975, PMID: 32581909 PMC7290241

[ref28] Wang P. Su M. Wang J. (2021). Organized crime in cyberspace: how traditional organized criminal groups exploit the online peer-to-peer lending market in China. Br. J. Criminol. 61, 303–324. doi: 10.1093/bjc/azaa064

[ref29] Wu Y. Song D. (2019). Gratifications for social media use in entrepreneurship courses: learners' perspective. Front. Psychol. 10:1270. doi: 10.3389/fpsyg.2019.01270, PMID: 31214081 PMC6555126

[ref30] Wu J. Wu L. (2019). Evaluation of medical college Students' entrepreneurial skills and its relationship with social intelligence. Open J. Soc. Sci. 7, 13–23. doi: 10.4236/jss.2019.74002

[ref31] Yang Q. Wang Y. Ren Y. (2019). Research on financial risk management model of internet supply chain based on data science. Cogn. Syst. Res. 56, 50–55. doi: 10.1016/j.cogsys.2019.02.001

[ref32] Zhao J. Wei G. Chen K. H. Yien J. M. (2020). Psychological capital and university students' entrepreneurial intention in China: mediation effect of entrepreneurial capitals. Front. Psychol. 10:2984. doi: 10.3389/fpsyg.2019.02984, PMID: 32038375 PMC6989491

